# Prevalence of dental caries in children with congenital heart disease

**DOI:** 10.1186/s12887-022-03769-2

**Published:** 2022-12-12

**Authors:** Steffen Koerdt, Julia Hartz, Stefan Hollatz, Max Heiland, Norbert Neckel, Peter Ewert, Renate Oberhoffer, Herbert Deppe

**Affiliations:** 1 Department of Oral and Maxillofacial Surgery, Corporate Member of Freie Universität Berlin, Charité – Universitätsmedizin Berlin, Humboldt-Universität zu Berlin, and Berlin Institute of Health, Augustenburger Platz 1, 13353 Berlin, Germany; 2 Department of Oral and Maxillofacial Surgery, Technical University of Munich (TUM), Ismaninger Str. 22, D-81675 Munich, Germany; 3 Department of Sport and Health Sciences, Chair of preventive pediatrics, Technical University of Munich (TUM), Georg-Brauchle-Ring 60/62, D-80992 Munich, Germany; 4 Department of Pediatric Cardiology and Congenital Heart Defects, German Heart Center, Technical University of Munich (TUM), Lazarettstraße 36, D-80636 Munich, Germany

**Keywords:** Infective endocarditis, congenital heart disease, pediatrics, oral health, dmft, caries

## Abstract

**Background:**

Congenital heart defects (CHD) affect about 8 out of 1000 births worldwide. Most of the patients reach adulthood and are exposed to an increased risk of endocarditis. Since bacteria already enter the bloodstream during everyday activities, oral hygiene is given special importance in the prevention of endocarditis.

**Methods:**

In this study 81 boys (55.1%) and 66 (44.9%) girls with CHD received a dental exam and additionally an assessment using the DIAGNOdent® pen. This study group consisting of patients with CHD was matched with a healthy epidemiological control group in Germany.

**Results:**

Eighty-one boys (55.1%) and 66 (44.9%) girls were examined. The mean age was 11 ± 4 years.

38.8% showed at least one untreated carious lesions. 37.4% had a dmft/DMFT ≥2 and thus represented a group with an increased caries risk.

The dmft value was 2.12 ± 1.25 in the age group 3-6 year olds. In the group of the 7-12 year old patients the DMFT/dmft was 2.06 ± 2.27, whereas DMFT in 13-17 year olds was at 2.12 ± 1.58. However, children and adolescents with CHD had a higher DMF index than healthy children in the same age group.

**Conclusions:**

The present study reveals that more than one third of those examined have a dental condition in need of rehabilitation. In future, close interdisciplinary cooperation between pediatric cardiologists and dentists should ensure regular dental check-ups.

## Background

The incidence of congenital heart diseases (CHD) is reported to be approximately 8-10 cases per 1000 live births. Complex anomalies account for almost one-third of all children with CHD [1]. However, innovations and progress in the field of pediatric cardiac surgery as well as anesthesiology have made it common for children with CHD to reach adulthood. Therefore, dental health becomes more and more important as CHD represents a predisposing cardiac condition for the development of infective endocarditis (IE) [2]. Studies from the literature have shown that approximately 15% of IE cases were caused by oral bacteria and occurred after a recent dental treatment [3, 4]. Dental health and maintenance of an excellent dental hygiene is especially important in this specific patient collective. For example, many of the children suffering from CHD have difficulties with nutrition during their first years of life. Frequent feeds and night meals are sometimes necessary to maintain a sufficient level of calorie intake. In addition, some drugs contain sugar and together with diuretics are known to cause xerostomia [5]. An increased caries prevalence and more untreated caries were shown in previous studies [4]. A study by Stecksén-Blicks et al. investigating 41 children with CHD compared to a healthy control group revealed a significantly increased caries prevalence in spite of intensive preventive efforts [6]. DMFS/dmfs values as well as bitewing radiographs were used for assessment. Pollard and coworkers found *streptococcus mutans* to be the leading pathogen in saliva of CHD children and to be positively correlated to the number of decayed teeth [4]. Our own study group evaluated the knowledge about the connection between IE and CHD in parents of CHD children. Dental prevention and disease awareness are still subject to improvement even in educated communities [7].

However, a non-invasive examination seems of special importance in this patient collective, as even small procedures such as periodontal probing might already cause bacteremia and increase the risk for the onset of IE. The DIAGNOdent® pen (KaVo, Germany) using laser fluorescence technology respresents an addiontal non-invasive toll in assessment of dental caries [8]. The DIAGNOdent® pen has been proven to be an appropriate tool for routine examinations in children [9, 10]. This is especially true for proximal and occlusal tooth surfaces [11, 12]. Therefore, the use of the DIAGNOdent® pen seems just a consequent development in this vulnerable group of patients.

The aim of the current study was to assess the caries prevalence in a group of children suffering from CHD compared with age and gender matched pairs from community examinations. Moreover, the use of DIAGNOdent® pen as an additional diagnostic tool in this clinical setting was evaluated.

## Methods

### Patients

A total of 147 children with different types of CHD were included in the current study. All children were patients, either in the outpatient department or on the ward at the Department of Pediatric Cardiology and Congenital Heart Defects at the German Heart Centre Munich of the State of Bavaria and the Technical University of Munich, Germany between 1st of June 2016 and 31st of March 2017. All patients with CHD were included, while children with cardiac dysrhythmia were excluded. CHD were categorized according to the definition of Warnes et al. (Table 1) [13]. A healthy study collective was obtained from records of community dental exams in the federal state of Saxonia, Germany. Examinations from 2015/2016 were performed at 3 to 17 year old children. However, no primary teeth were included after the age of 10, according to recommendations of the federal association of community dentist in Germany (BZÖG) [14]. The presented study was approved by The Ethics Committee of the Technical University of Munich (133/16S. Informed consent was obtained from all legal guardians. All methods were performed in accordance with relevant guidelines and regulations.

**Table 1:** Classification of congential heart disease (CHD) according to Warnes et al. [3] and their incidence in the current study

Category	Complexity	Types of patients
	n (%)	
I.	**great**	Conduits, valved or nonvalved
	**68 (45.3)**	
		Cyanotic congenital heart (all forms)
		Double-outlet ventricle
		Eisenmenger syndrome
		Fontan procedure
		Mitral atresia
		Single ventricle (also called double inlet or outlet, common or primitive)
		Pulmonary atresia (all forms)
		Pulmonary vascular obstructive diseases
		Transposition of the great arteries
		Tricuspid atresia
		Truncus arteriosus/hemitruncus
		Other abnormalities of atrioventricular or ventriculoarterial connection not included above (i.e., crisscross heart, isomerism, heterotaxy syndromes, ventricular inversion)
II.	**moderate**	Aorto-left ventricular fistulae
	**64 (42.7)**	
		Anomalous pulmonary venous drainage, partial or total
		Atrioventricular canal defects (partial or complete)
		Coarctation of the aorta
		Ebstein’s anomaly
		Infundibular right ventricular outflow obstruction of significance
		Ostium primum atrial septal defect
		Patent ductus arteriosus (not closed)
		Pulmonary valve regurgitation (moderate to severe)
		Pulmonic valve stenosis (moderate to severe)
		Sinus of Valsalva fistula/aneurysm
		Sinus venosus atrial septal defect
		Subvalvar or supravalvar aortic stenosis (except HOCM)
		Tetralogy of Fallot
		Ventricular septal defect with
		Absent valve or valves
		Aortic regurgitation
		Coarctation of the aorta
		Mitral disease
		Right ventricular outflow tract obstruction
		Straddling tricuspid/mitral valve
		Subaortic stenosis
III.	**simple**	Native disease
	**18 (12)**	
		Isolated congenital aortic valve disease
		Isolated congenital mitral valve disease (e.g., except parachute valve, cleft leaflet)
		Isolated patent foramen ovale or small atrial septal defect
		Isolated small ventricular septal defect (no associated lesions)
		Mild pulmonic stenosis
		Repaired conditions
		Previously ligated or occluded ductus arteriosus
		Repaired secundum or sinus venosus atrial septal defect without residua
		Repaired ventricular septal defect without residua

### Study design

The study was designed according to the guidelines of the declaration of Helsinki. The prospective study was approved by the local ethics committee of the Technical University of Munich (133/16S). All parents of the patients were informed extensively and gave their informed consent.

### Dental examination

Parents were informed, that the participation in this study does not replace a visit to the pediatric dentist. However, in cases when the need for an urgent dental intervention was detected during the study examination, the parents were informed and advised to see their dentist. The dental exams were performed by the same investigator throughout the study to avoid individual effects and influences during the dental examination. DMFT and dmft scores, representing decayed (D/d), missing (M/m), and filled teeth (F/f) accounting for dental caries prevalence as well as dental treatment needs, were assessed. Caries was only accounted positive if definite (D3-D4). Initial caries was not counted as positive. Missing teeth in transitional dentition were only counted as missing if extracted for pathological cause. Extractions for orthodontic treatment were documented but not counted as missing.

### Examination using the DIAGNOdent® pen

DIAGNOdent® pen 2190 (KaVo, Germany) is registered as a medical product (93/42/EWG and 2004/108/EG) and used as a laser class I (IEC 60825-1:1993 + A1:1997+ A2:2001). Laser induced fluorescence between healthy hard tooth tissue and caries is converted into numerical values. These can be used as an additional parameter in defining risk factors for dental caries. Table 3 summarizes reference values from the literature and gives guidance of further diagnistics and treatment according to the specific values (Table 3). Assessment using the DIAGNOdent® pen was performed in sextants as displayed in Fig. 2.

### Statistical analysis

All data were analyzed by using IBM® SPSS® for Mac (version 26.0; IBM Corp., USA). Means and standard deviation (SD) were calculated, and tests of significance were performed. For normally distributed values, t-test was performed. For values not normally distributed, the Mann–Whitney and Kruskal-Wallis tests were used. Statistical significance was defined as *α* = 0.05.

## Results

Eighty-one (55.1%) male and 66 (44.9%) female patients were enrolled in the current study. 91 (62%) were outpatients, whereas 56 (38%) patients were treated as inpatients on pediatric wards at the German Heart Center. The mean age was 11 ± 4 years. Distribution in age groups (primary, changing, and permanent dentition) was as follows: 33 (22.4%) 3-6 year olds; 63 (42.9%) 7-12 year olds; and 51 (34.7%) 13-17 year olds. The dmft value was 2.12 ± 1.25 in the age group 3-6 year olds. In the group of the 7-12 year old patients the DMFT/dmft was 2.06 ± 2.27, whereas DMFT in 13-17 year olds was at 2.12 ± 1.58. However, dmft, dmft/DMFT and DMFT values respectively were all lower within the healthy study collective (Table 2). Nevertheless, differences were only statistically significant in age groups 7-12 (*p* = 0.009) and in 13-17 year olds (*p* = 0.001).

**Table 2:** Comparison of DMFT/dmft values in different age groups in children with CHD and healthy controls

Age group	n	Mean		*p*-value *
		*Controls*	*CHD*	
3-6	33	0.99	2.12	0.068
7-12	63	1.26	2.06	0.009
13-17	51	0.93	2.12	0.001

Abbreviations: *CHD* Congenital Heart Disease, * *t* test

Taking only the result from the dental examinations in CHD children into account, a few surprising facts concerning general dental health become obvious. Ninety children with CHD (61.5%) were free of caries and showed an excellent oral hygiene. However, a total of 57 children suffering from CHD (38.87%) showed at least one untreated dental lesion. Fifty-five patients (37.4%) showed an increased dmft/DMFT value of 2 or larger and represent a group of patients with an increased caries level.

**Fig. 1:**
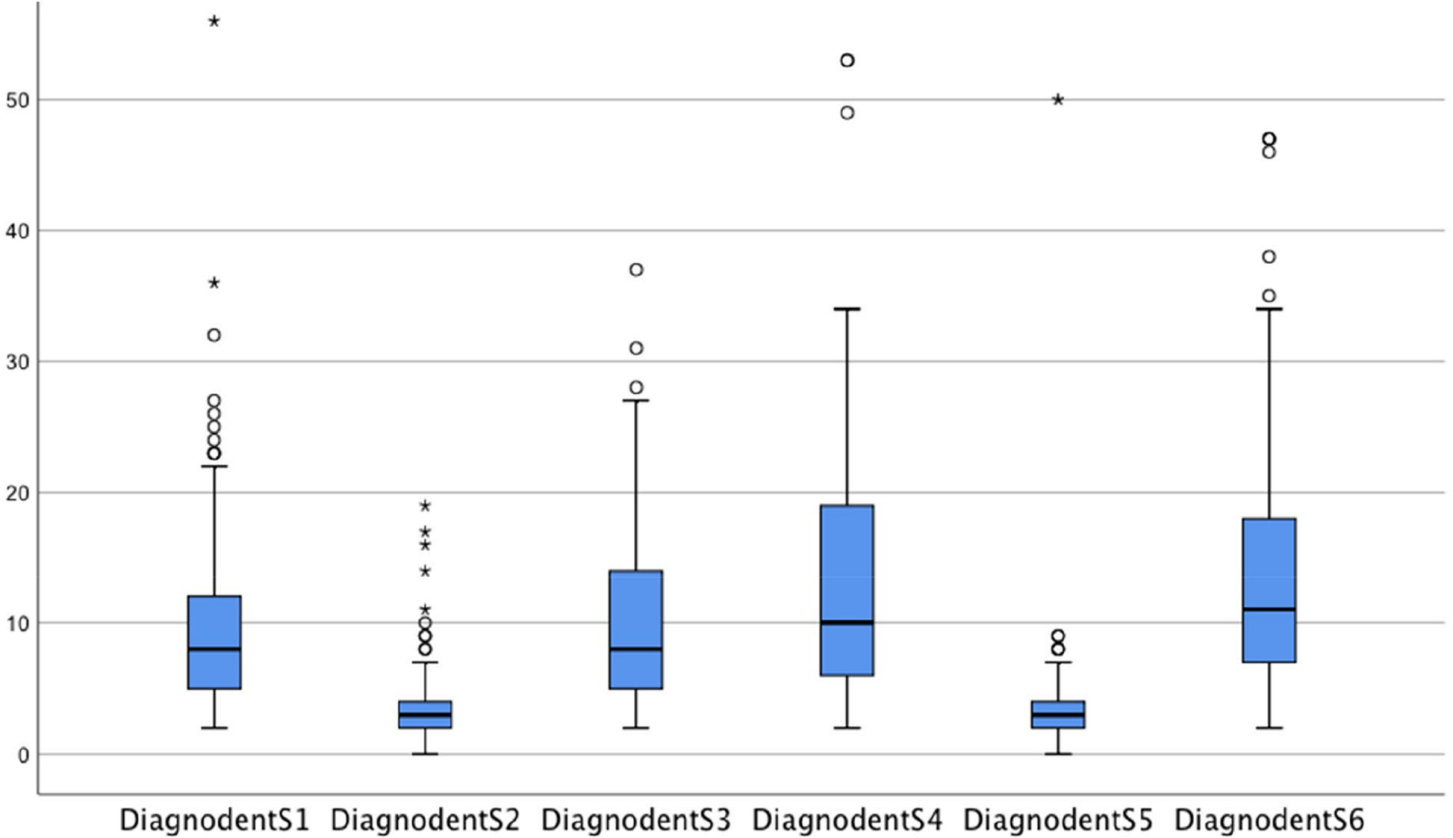
Boxplot diagrams visualizing the results of DIAGNOdent® pen measurements in CHD children. Median values (with 75th and 25th percentiles) are displayed. The range is shown as a vertical line; extreme values are included

**Fig. 2:**
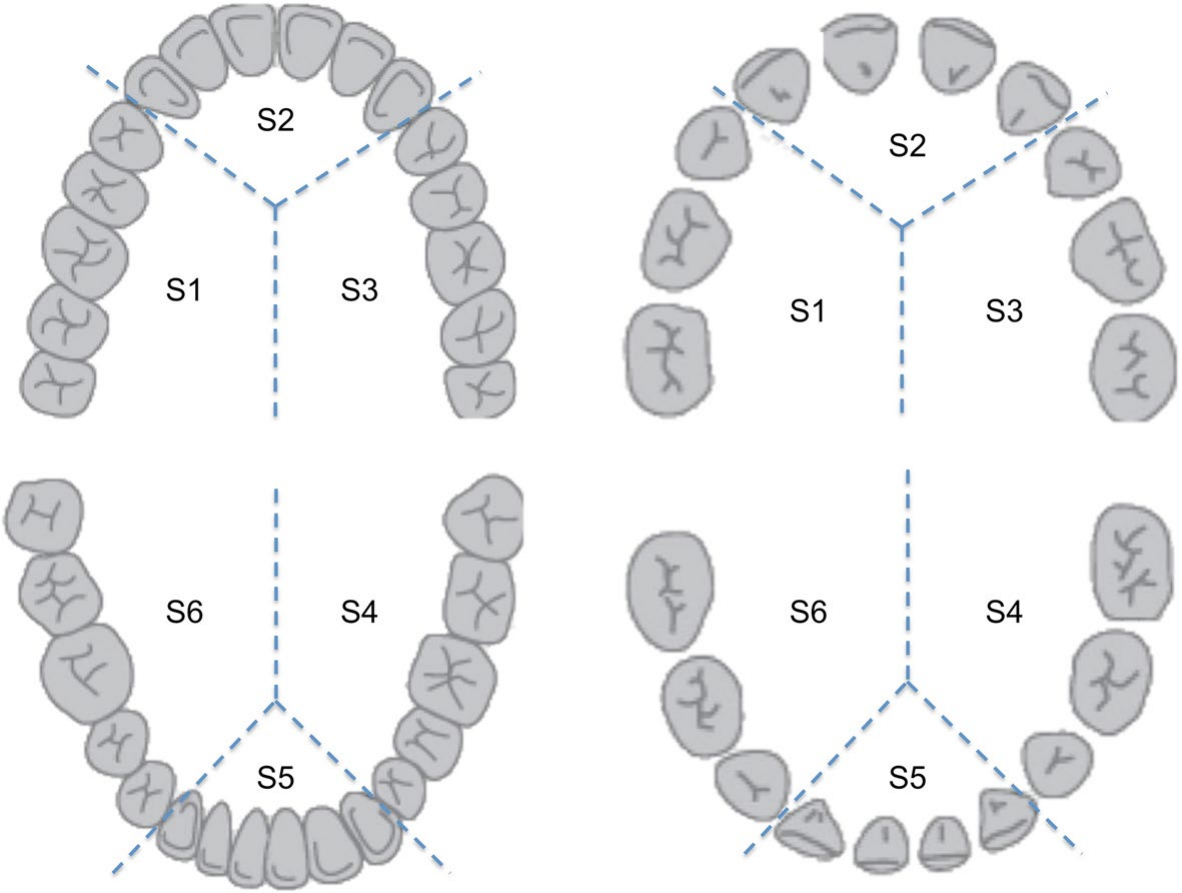
Sextants counting clockwise as used for evaluation of DIAGNOdent® pen examination in primary (right) and permanent dentition (left)

The results of the 12 examinations using the DIAGNOdent® are displayed in Fig. 1, whereas numerical values were measured in sextants counting clockwise (Fig. 2). Front teeth appear to have smaller values than posterior teeth, whereas the cut-off value has been defined with ≥21. Reference values from the literature are given in Table 3.

**Table 3:** Cut-off values of the DIAGNOdent® pen (modified [11, 12])

Type of caries	none	fissural		
value	0 – 13	14 – 20	21 – 29	> 30
clinical diagnosis	healthy tooth structure	enamel caries	deep enamel caries	dentine caries
measures	–	monitoring	caries risk analysis	x-ray
			saliva&bacteria testing	
actions	prophylaxis (e.g. fluoridated toothpaste)	intensified prophylaxis (e.g. fluoridation)	if low risk: intensified prophylaxis	minimalinvasive treatment
			if high risk: minimalinvasive treatment	

## Discussion

Dental health and oral hygiene seem to be especially important in patients and children who suffer from congenital heart disease (CHD), as bacteremia, and even the transient occurrence of bacteria through everyday life procedures as brushing teeth or even flossing are connected with a higher risk for infective endocarditis (IE) [15]. Dental health and the prevention of invasive dental treatments have therefore to be of special importance. The close connection between IE and CHD and respectively the need for the maintenance of an excellent oral hygiene is a key factor in the complex pathognomonic network associated with CHD [16].

The risk for patients with a CHD to suffer from an IE is significantly increased and despite optimal care, mortality approaches 30% at 1 year [17]. However, the risk for a lethal course of an IE is considered to be the greatest in patients with congenital cyanotic defects and in patients up to 6 months after operative or interventional repair using alloplastic material, or lifelong if a residual shunt or valve insufficiency. These patients are high-risk patients and an antibiotic prophylaxis is recommended before dental treatment. Current guidelines advise a prophylaxis in high-risk patients with CHD as mentioned above as well as patients with valve prothesis or reconstructed valves using alloplastic materials, and patients who already suffered from an IE using amoxicillin or clindamycin if an allergy is known [18, 19]. This emphasizes the importance of an excellent oral health especially in CHD patients and even more important in children. A case-control study by Cantekin and coworkers from 2013 investigated the dental health of 268 CHD patients compared with a healthy control group. Significant differences between the caries experience, the oral health status, and the dental age of children with CHD were found compared with healthy children [20]. However, social factors such as the home country and the access to dental preventive facilities seem also to play an important role in evaluating dental health in CHD patients [21, 22].

In addition to the increased risk for the development of IE in CHD patients, oral health and worries about the maintance of an excellent oral hygiene and the prevention of complications associated with IE, also seem to have an impact on the quality of life of these young patients and their parents as da Fonseca et al. were able to show [23].

Nevertheless, the use of a non-invasive and easy to use tool such as DIAGNOdent® pen has not been evaluated in dental screening examinations of CHD children. This current study revealed that in the CHD study collective a percentage of 36.7% of all patients had no history of dental caries (dmft/DMFT = 0), although almost the same number of participants (37.4%) an increased caries level with dmft/DMFT-values > 2. The mean dmft/DMFT value was 2.28 ± 2.69. In a detailed analysis of the single components of the DMF-index it becomes obvious that even though 60.7% of all patients are caries free (d/D = 0), but according to Balmer et al. 38% have at least one untreated caries lesion [24]. These findings are consistent with reports from the literature as Sivertsen et al. report that one third of all patients with CHD have a dental condition, that might potentially harm the general health [25]. However, the prevalence of caries in children and teenagers does not show a normal curve of distribution, DMF-means and standard deviations do not fully reflect the impact and extent of the disease [26]. The Significant Caries Index (SiC-Index) as introduced by Bratthall et al. in this current study is 5.41, and almost twice as large as proposed by the WHO for 12-year old children in 2015. However, this current study could not reveal any correlation between the severity of the underlying CHD and either the SiC or dmft/DMFT Index. Besides a social component, the general health of patients with the same complexity of their heart defect changes a lot (Table 1). The number of operative interventions and the frequency and duration of in-hospital stays surely influence the general and even oral health. In comparison of high- and low-risk patients concerning the indication for antibiotic prophylaxis results also were significant. This is surprising, as especially patient from the high-risk group were more frequently informed about the connection between IE and oral hygiene.

The results of the examination using the DIAGNOdent® Pen can however only be interpreted with limitations and represent just an additional non-invasive tool that might support clinical findings from a sufficient oral exam and should not be depicted separately.

In general, a few conception aspects in study design should be mentioned and kept in mind when discussing the study results. No professional tooth cleaning was performed before the use of the DIAGNOdent® pen, due to the examinations on the pediatric wards. DIAGNOdent® results should therefore be considered as a tool to support an informative basis by a substaintiated dental clinical examination. Moreover, dmft/DMFT was just determined in a visual exam, as a sharp testing probe could potentially harm the occlusal relief [27]. As only active participants were included in the study, non-responders, meaning children with especially bad dmft/DMFT values were not included. Therefore, dmft/DMFT values as described are probably lower than in reality [28].

In summary, this current study aims to give an overview of the prevalence of dental caries in children and teenagers with CHD in an industrialized country such as Germany. The results show, that children and teenagers with CHD surprisingly have a significantly increased prevalence of caries and a worse oral hygiene. The reasons for this as outlined above are complex and manifold. However, the results of this study call for better information and education of parents and community dentists as well as pediatric cardiologists alike. As antibiotic prophylaxis is only one central column in the complex dental treatment of CHD patients, even more important is an early and consistent prophylaxis of any invasive treatment. This can only be achieved with a close escort of the parents and the children. Therefore, community and family dentists should be aware of the general health conditions even of their youngest patients and under the correct circumstances should not hesitate to perform even invasive dental treatment despite the underlying CHD to avoid aggravation and worsening of the dental and general health. Nevertheless, this study only represents a small cross-sectional analysis in Germany. For a comprehensive evaluation multicenter studies over a sufficient period of time are needed.

## Strengths and limitations

This current study has a few limitations, that should be kept in mind when interpreting the results. First of all the use of the DIAGNOdent® pen without a previous professional tooth cleaning, the use on a pediatric ward and not in a professional dental set up, a selection bias of patients in the study group, as well as the waiving of sharp probing in order to avoid bleeding. Moreover, the sample size is low and represents only a snap-shot. Nevertheless, this study is able to introduce the DIAGNOdent® pen as an easy device in dental assessment, especially in this specific group of patients.

## Conclusions

The current study is able to show, that Children and teenagers with CHD surprisingly have a significantly increased prevalence of caries and a worse oral hygiene than a healthy collective despite their need for an excellent oral health in order to prevent the onset of infective endocarditis. Moreover, the use of the DIAGNOdent® pen for initial an non-invasive dental assessment especially in this specific group of patients has been established. Consequently, pediatric dentist should help to improve the information and education of parents and community dentists as well as pediatric cardiologists alike and intensivy screening methods using tools such as DIAGNOdent®.

### Additional declarations for articles in life science journals that report the results of studies involving humans and/or animals

This study complies with the STROBE criteria for observational studies as well as the declaration of Helsinki.

## Data Availability

The datasets analysed during the current study are not publicly available as they have not been published but are available from the corresponding author on reasonable request.
